# Assessment of non-tuberculosis abnormalities on digital chest x-rays with high CAD4TB scores from a tuberculosis prevalence survey in Zambia and South Africa

**DOI:** 10.1186/s12879-023-08460-0

**Published:** 2023-08-08

**Authors:** Dennis Ngosa, Given Moonga, Kwame Shanaube, Choolwe Jacobs, Maria Ruperez, Nkatya Kasese, Eveline Klinkenberg, Ab Schaap, Linda Mureithi, Sian Floyd, Sarah Fidler, Veronica Sichizya, Adrian Maleya, Helen Ayles

**Affiliations:** 1 Department of Epidemiology and Biostatistics, School of Public Health, The University of Zambia, Lusaka, Zambia; 2 Zambia Aids Related Tuberculosis (ZAMBART), Lusaka, Zambia; 3 London School of Hygiene and Tropical Medicine, London, UK; 4 Department of Global Health, Amsterdam University Medical Centers, Amsterdam, the Netherlands; 5 Health Systems Trust, Cape Town, South Africa; 6 Department of Infectious Disease, Faculty of Medicine, Imperial College London, London, UK; 7 University Teaching Hospital, Lusaka, Zambia; 8 Apex Medical University, Lusaka, Zambia

**Keywords:** Prevalence, Computer-aided detection, Non-TB abnormalities, Digital chest X-rays

## Abstract

**Background:**

Chest X-rays (CXRs) have traditionally been used to aid the diagnosis of TB-suggestive abnormalities. Using Computer-Aided Detection (CAD) algorithms, TB risk is quantified to assist with diagnostics. However, CXRs capture all other structural abnormalities. Identification of non-TB abnormalities in individuals with CXRs that have high CAD scores but don’t have bacteriologically confirmed TB is unknown. This presents a missed opportunity of extending novel CAD systems’ potential to simultaneously provide information on other non-TB abnormalities alongside TB. This study aimed to characterize and estimate the prevalence of non-TB abnormalities on digital CXRs with high CAD4TB scores from a TB prevalence survey in Zambia and South Africa.

**Methodology:**

This was a cross-sectional analysis of clinical data of participants from the TREATS TB prevalence survey conducted in 21 communities in Zambia and South Africa. The study included individuals aged ≥ 15 years who had high CAD4TB scores (score ≥ 70), but had no bacteriologically confirmed TB in any of the samples submitted, were not on TB treatment, and had no history of TB. Two consultant radiologists reviewed the images for non-TB abnormalities.

**Results:**

Of the 525 CXRs reviewed, 46.7% (245/525) images were reported to have non-TB abnormalities. About 11.43% (28/245) images had multiple non-TB abnormalities, while 88.67% (217/245) had a single non-TB abnormality. The readers had a fair inter-rater agreement (*r* = 0.40). Based on anatomical location, non-TB abnormalities in the lung parenchyma (19%) were the most prevalent, followed by Pleura (15.4%), then heart & great vessels (6.1%) abnormalities. Pleural effusion/thickening/calcification (8.8%) and cardiomegaly (5%) were the most prevalent non-TB abnormalities. Prevalence of (2.7%) for pneumonia not typical of pulmonary TB and (2.1%) mass/nodules (benign/ malignant) were also reported.

**Conclusion:**

A wide range of non-TB abnormalities can be identified on digital CXRs among individuals with high CAD4TB scores but don’t have bacteriologically confirmed TB. Adaptation of AI systems like CAD4TB as a tool to simultaneously identify other causes of abnormal CXRs alongside TB can be interesting and useful in non-faculty-based screening programs to better link cases to appropriate care.

**Supplementary Information:**

The online version contains supplementary material available at 10.1186/s12879-023-08460-0.

## Introduction

Before the coronavirus (COVID-19) pandemic, tuberculosis (TB) had surpassed HIV/AIDS as the most common infectious cause of death worldwide [1]. In 2021, Africa accounted for 23% of world TB incidence with South Africa (SA) falling among the top 8 while Zambia fell among the top 30 countries the with highest TB burden globally [1]. Countries have been adopting various strategies to try and reduce the burden of TB in a move to meet the end TB strategy by 2030 [2]. Despite this, the TB care cascade has been facing numerous challenges, including case detection related to missed diagnosis and late detection [3, 4]. As a result, the diagnostic algorithm for TB in TB-related programs such as community-based screening and active case finding (ACF) has attempted to use diagnostic tools that are highly sensitive to TB to increase the case detection rate [3, 4].

In tuberculosis prevalence surveys (TBPS), chest X-rays (CXRs) have traditionally been used in the primary detection of TB-suggestive abnormalities alongside symptoms with subsequent GeneXpert/RIF and bacteriological culture tests [5]. In recent years, there has been renewed interest in the use of CXRs due to the new method of using artificial intelligence (AI) such as Computer Aided Detection (CAD) systems to read for presumptive TB abnormalities to supplement the traditional method of letting humans (clinicians) read for abnormalities indicative of TB [6]. Computer Aided Detection systems work by producing a score on each digital CXR. Digital CXRs with CAD scores above a set threshold (high score) suggest a likelihood of TB, while those with CAD scores below that set threshold (low score) suggest the unlikelihood of TB [7, 8]. This method of using digital CXRs with CAD systems to read for abnormalities indicative of TB has been on the rise, especially in low-to-middle income countries (LMICs) like Zambia and SA with limited human resources of qualified radiologists and other clinical specialists properly trained to read digital CXRs [6], and when large numbers of CXRs need to be read. i.e., in a prevalence survey (PS) or ACF setting.

During TB screening activities, CAD systems such as CAD4TB (Delft Imaging, the Netherlands) [9] are programmed to read for the likelihood of TB on digital CXRs [10]. Even though this is the case, not everyone with a digital CXR that has a high CAD4TB score (indicating an abnormality) turns out as a confirmed TB case (GeneXpert/RIF and/or bacteriological culture based) [11–14]. Digital CXRs with CAD4TB systems have a high sensitivity for TB (ranging from 90 to 100%) but a relatively low specificity (ranging from 23 to 58%) [11–14]. Previous studies have further shown that digital CXRs can demonstrate other abnormalities which might indicate the presence not only of TB but also other communicable and/or non-communicable diseases like chronic respiratory diseases and cardiovascular diseases [15–19].

Apart from being among the top 30 countries with the highest TB burden globally [1], Zambia and SA are also countries in Southern Africa that have a high burden of non-TB abnormalities (cardiovascular and pulmonary conditions) like lung cancers [20, 21], and idiopathic cardiomegaly [22], among others. Although South Africa has been reported to have a more dynamic picture of non-TB abnormalities such as pleural effusions related to TB and pneumonia [23] as well as silicosis which has also been documented to be associated with TB [24].

Studies that have been done on CXRs in other Sub-Saharan African countries have reported a high prevalence of non-TB abnormalities such as cardiomegaly with heart failure, chronic obstructive lung disease (COPD), and post-TB lung changes [15, 16]. These studies investigated non-TB abnormalities on all abnormal CXRs irrespective of the CAD scores and TB history. However, specific non-TB abnormalities that can be identified in a sub-group of individuals with digital CXRs that have high CAD scores yet no bacteriologically confirmed TB and no history of TB are not well known due to limited literature.

This presents a missed opportunity in utilizing novel CAD systems’ potential to simultaneously provide information on other clinically relevant conditions in communities alongside TB. Therefore, the objective of this study was to characterize and estimate the prevalence of non-TB abnormalities on digital CXRs with high CAD4TB scores from a TB prevalence survey in selected communities in Zambia and South Africa.

## Methods

### Study design and population

This was a nested cross-sectional analysis of clinical data from the TREATS (Tuberculosis Reduction through Expanded Antiretroviral Treatment and Screening) TBPS from 12 peri-urban communities in Zambia and 9 from the Western Cape province of SA.

The TBPS which was conducted from 2018–2021, measured the prevalence of TB in a randomly selected sample of ~50,000 people aged ≥ 15 years and it was one of the studies under the TREATS project (reported elsewhere) [25].

Overall, the TREATS project measured the impact of a combined TB/HIV preventive intervention of population-level screening for TB, combined with universal testing and treatment (UTT) for HIV on notified TB incidence, prevalence of TB disease, and incidence of TB infection in Zambia and SA (reported elsewhere) [25, 26].

### Inclusion criteria

The study included participants aged 15 years and above, residents in the selected study communities who took part in the TREATS TBPS and had high CAD4TB scores based on outputs from CAD4TB version 5.0 (Delft Imaging, the Netherlands) [9]. A high CAD4TB score was defined as a score of 70 and above. This was adopted from the TREATS TBPS protocol which was guided by the pilot study that was done in 2018 [25, 27].

### Exclusion criteria

All participants who were found to have bacteriologically confirmed TB during the TBPS were excluded from the study. TB was defined as either positive Xpert-Ultra results (low, medium, or high) or positive bacteriological culture test results (*Mycobacterium tuberculosis*). Participants with *non-tuberculosis mycobacterium* (*NTM*) or trace results were also excluded from the study. The study also excluded all participants who reported a history of TB as well as all participants who reported being on TB treatment at the time of participating in the TBPS. The primary focus of the current study was non-TB abnormalities on CXRs with high CAD4TB scores. Hence, to avoid having most of the CXRs being read for suspected TB or post-TB lung changes, the study adopted the above inclusion and exclusion criteria.

### Study procedures

Digital CXRs with CAD4TB scores and baseline characteristics of individuals who met the selection criteria were extracted from the TREATS TBPS database. A separate online archive database containing the digital CXRs of the selected participants was set up for reading and reporting, with two separate accounts for consultant radiologists 1 and 2. Both radiologists had approximately 20 years of experience as consultant radiologists, one of them was based at University Teaching Hospital (UTH), while the other one was based at Apex Medical University, Lusaka Zambia.

#### Image reading

After being given access to the online database, the radiologists were oriented to the reading and reporting system. The radiologists were blinded from each other’s readings and CAD4TB scores. A pilot test was done on 20 images (separate images from the main study) to test the credibility of the reading and reporting tools. The two radiologists then conducted the main reading and reporting of non-TB abnormalities identified on digital CXRs. Each radiologist read and reported on all images separately according to a reporting tool adopted from Fleischner Society guidelines [15, 28] and modified according to the requirements of this study. All the readings were recorded and stored on the online reading and reporting system.

### Statistical analysis

All CAD4TB scores on CXRs were produced using CAD4TB version 5.0 of Delft imaging. Data were analyzed using STATA version 14.0 software. The baseline characteristics of participants were summarised using descriptive statistics.

Inter-rater agreement (IRA) was calculated using the Cohens kappa statistic index [29, 30]. The Cohens kappa values were interpreted as follows; < 0.00 = poor agreement, 0.00–0.20 = slight agreement, 0.21–0.40 = fair agreement, 0.41–0.60 = moderate agreement, 0.61–0.80 = substantial agreement and 0.81–1.00 = almost perfect agreement as proposed by Landis and Koch [31].

The outcome variable was non-TB abnormalities. Prevalences of non-TB abnormalities with 95% confidence intervals (CI) were calculated and presented for all primary non-TB abnormalities and also for grouped non-TB abnormalities characterized according to 4 anatomical regions, based on the location of the abnormalities in the chest area. The denominator for prevalence calculations was 525 which was the study sample size. All the analyses in this study were done at 95% confidence interval.

## Results

### Description of the study population

**Fig. 1:**
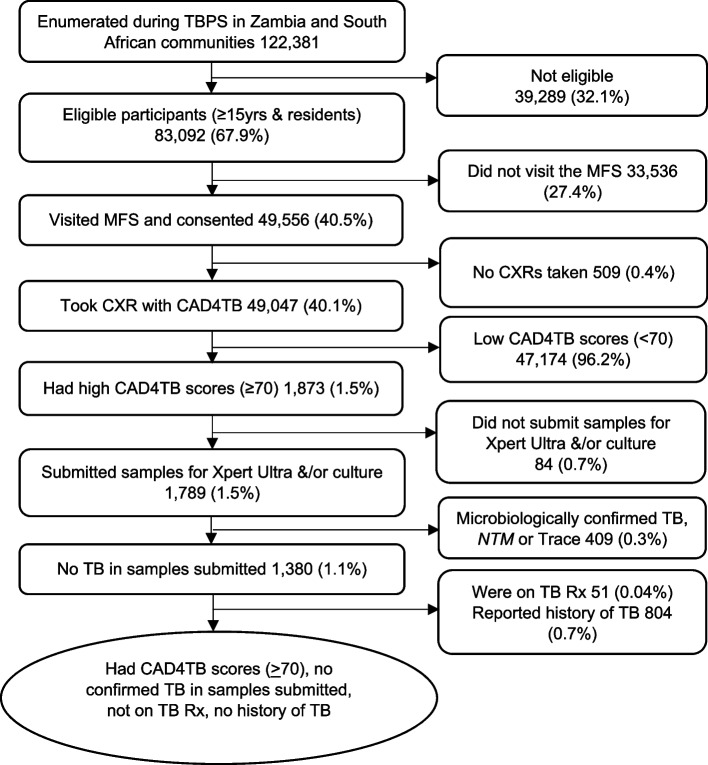
Study Flow diagram. Rx = Treatment, TB = Tuberculosis, PS = Prevalence survey, CAD4TB = Computer-aided detection for tuberculosis, MFS = Mobile filed site

During the TREATS TBPS, a total of 122,381 individuals were enumerated from both Zambia and SA communities. Out of these, 83,092 (67.9%) participants were eligible (≥ 15 years and residents in the communities) to take part in the TBPS. A total of 49, 556 (40.5%) participants visited the mobile field sites (MFS) and 49,047 (40.1%) had digital CXRs taken and scored using the CAD4TB version 5 system. However, only 1,873 (1.5%) participants had high CAD4TB scores (≥ 70). About 1,789 (1.5%) participants submitted samples for microbiological TB confirmation (Xpert Ultra and/or culture test) and 1,380 (1.1%) participants had no TB detected in any of the samples submitted. The total sample that was used in this study was 525 (0.4%), which represented participants that had high CAD4TB scores but no TB detected in any of the samples submitted, were not on TB treatment at the time of participating in the TBPS and reported no history of TB (Fig. 1).

### Baseline characteristics of participants

Out of 525 participants that were included in this study, 52.6% (276/525) came from Zambian communities while 47.4% (249/525) came from South African communities. Approximately half of the participants 51.6% (271/525) were males and the median age was 46 (IQR 33–61). The median CAD4TB score was 81 (IQR 73–92) and half of the participants 49.3% (259/525) had CAD4TB scores ranging between 70–80, while less than a quarter 22.1% (16/525) had CAD4TB scores ranging between 81–90 and more than a quarter 28.5% (150/525) had CAD4TB scores ranging between 91–100 (Table 1).

**Table 1:** Image reading and characteristics of participants with high CAD4TB scores from TREATS TB prevalence survey

**Characteristics**	**Total n (%)**	**Image reading n (%)**		***P* value**
		**No non-TB abnormalities**	**Suspected non-TB abnormalities**	
**Overall**	525	280 (53.3)	245 (46.7)	
**Sex**				
Male	271 (51.6)	142 (52.40)	129 (47.60)	0.657^C^
Female	254 (48.4)	138 (54.33)	116 (45.67)	
**Age**				
(M/IQR)	46 (33–61)	46.5 (32.5–60)	46 (34–62)	0.478^R^
**Reported cough**				
Yes	84 (16.0)	45 (53.57)	39 (46.43)	0.962^C^
No	441 (84.0)	235 (53.29)	206 (46.71)	
**Reported weight loss**				
Yes	34 (6.5)	16 (47.06)	18 (52.94)	0.448^C^
No	491 (93.5)	264 (53.77)	227 (46.23)	
**Reported fever**				
Yes	26 (5.0)	15 (57.69)	11 (42.31)	0.648^C^
No	499 (95.0)	265 (53.11)	234 (46.89)	
**Reported night sweats**			-	
Yes	31 (5.9)	19 (12)	12 (38.71)	0.360^C^
No	494 (94.1)	261 (52.83)	233 (47.17)	
**Reported chest pains**				
Yes	52 (9.9)	29 (55.77)	23 (44.23)	0.711^C^
No	473 (90.1)	251 (53.07)	222 (46.93)	
**HIV status**				
Positive	83 (15.8)	44 (53.01)	39 (46.99)	0.509^C^
Negative	285 (54.3)	158 (55.44)	127 (44.56)	
Unknown	157 (29.9)	78 (49.68)	79 (50.32)	
**History of smoking**				
Yes	226 (43)	161 (53.85)	138 (46.15)	0.786^C^
No	299 (57)	119 (52.65)	107 (47.35)	
**Current alcohol drinkers**				
Yes	208 (39.6)	114 (54.81)	94 (45.19)	0.583^C^
No	317 (60.4)	166 (52.37)	151 (47.63)	
**CAD4TB score**				
(M/ IQR)	81 (73–92)	79 (73–89)	83 (74–94.5)	0.002^R^*
**CAD4TB score category**				
70–80	259 (49.3)	117 (45.17)	142 (54.83)	0.001^C^*
81–90	116 (22.1)	70 (60.34)	46 (39.66)	
91–100	150 (28.5)	93 (62.00)	57 (38.00)	
**Country**				
South Africa	249 (47.4)	137 (55.02)	112 (44.98)	0.462^C^
Zambia	276 (52.6)	143 (51.81)	133 (48.19)	

*n* Frequency, *%* Percentage, *M/IQR* Median and Interquartile range, *TB* Tuberculosis, *CAD4TB* Computer aided detection for tuberculosis, *C* Chi square test, *R* Wilcoxon ranksum test

^*^Statistically significant at 5% significance level

Furthermore, 43% (226/525) of clients reported a history of cigarette smoking and 39.5% (208/525) reported that they drank alcohol. Fifteen-point eight percent (83/525) of participants were HIV positive (Table 1).

### Inter-rater agreement (IRA) between reader 1 and reader 2

For the overall reading of non-TB abnormalities on digital CXRs, reader 1 and reader 2 had a slight inter-rater agreement with kappa = 0.18. Agreement based on the anatomical category of non-TB abnormality, the 2 readers had a fair inter-rater agreement for lung parenchyma, heart, and great vessels as well as mediastinum categories with kappa = 0.24, 0.30, and 0.33 respectively. While for the pleura category, the 2 readers had a slight inter-rater agreement with kappa = 0.13.

The 2 readers had a higher inter-rater agreement for non-TB abnormalities, lung parenchyma, mediastinum, and heart abnormalities for Zambian communities as compared to South African communities (Table 2).

**Table 2:** Inter-reader agreement between Reader 1 and Reader 2

**Level of agreement**	**Observed Agreement %**	**Expected Agreement %**	**Kappa**
**Overall reading**			
Non-TB abnormalities	62.86	54.59	0.18
***Anatomical category***			
Lung parenchyma	64.00	52.54	0.24
Pleura	59.43	53.57	0.13
Mediastinum	96.57	95.15	0.30
Heart	83.43	75.37	0.33
**Zambian communities only**			
Non-TB abnormalities	64.49	54.91	0.21
***Anatomical category***			
Lung parenchyma	66.67	52.54	0.30
Pleura	56.52	52.28	0.09
Mediastinum	97.10	95.74	0.32
Heart	84.78	74.64	0.40
**South African communities only**			
Non-TB abnormalities	61.04	54.18	0.15
***Anatomical category***			
Lung parenchyma	61.04	52.51	0.18
Pleura	62.65	55.13	0.17
Mediastinum	81.93	76.09	0.27
Heart	95.98	94.51	0.24

*TB* Tuberculosis

### Image reading and prevalence of non-TB abnormalities on digital CXRs with high CAD4TB scores

#### Image reading

Overall, both readers agreed on 46.7% (245/525) of images as having some form of non-TB abnormality. Among South African individuals, 44.98% (112/249) had non-TB abnormalities, while 48.19% (133/276) had non-TB abnormalities among individuals from Zambian communities. The median CAD4TB score for images with non-TB abnormalities was 83 (IQR 74–94.5), while for those without non-TB abnormalities was 79 (IQR 73–89) and this difference was statistically significant (*p* = 0.002) (Table 1).

From the 245 digital chest x-rays where both readers agreed as having some form of non-TB abnormality, 11.43% (28/245) images were reported to be having multiple non-TB abnormalities (2 or more) while 88.57% (217/245) images were reported to be having single non-Tb abnormalities.

#### Prevalence of non – TB abnormalities

For the prevalence of non-TB abnormalities where both readers agreed, pleural effusion/ thickening/ calcification was the most prevalent 8.8% (95% CI 6.5% - 11.5%). This was followed by cardiomegaly 5% (95% CI 3.3% - 7.2%). Other notable prevalences reported were pneumonia not typical of pulmonary TB and pleural thickening/calcification: likely benign where each had a prevalence of 2.7% (95% CI 1.5% -4.4%). Mass/nodules (benign/ malignant) and chronic scarring/volume loss, likely not related to TB where each had 2.1% (95% CI 1.1% - 3.7%) and interstitial patterns 1.5% (95% CI 0.7% - 3%) (Table 3).

**Table 3:** Prevalence of non-TB abnormalities on digital CXR with high CAD scores

**Category of non-TB abnormalities**	**Non-TB abnormality based on radiological diagnosis**	**Prevalence % (95% CI)**		
		**Only Reader 1** (discordant)	**Only Reader 2** (discordant)	**Both readers Agreed**
Lung parenchyma	Pneumonia not typical of PTB	6.7 (4.7–9.2)	5.2 (3.4–7.4)	2.7 (1.5–4.4)
	Chronic scarring/volume loss, likely not related to TB	6.3 (4.4–8.7)	1.1 (0.4–2.5)	2.1 (1.1–3.7)
	Mass/nodules: probably malignant/benign	6.3 (4.4–8.7)	1.1 (0.4–2.5)	2.1 (1.1–3.7)
	Interstitial pattern, other than edema	7.2 (5.2–9.8)	2.5 (1.3–4.2)	1.5 (0.7–3.0)
	Interstitial pattern from cardiac failure	3.8 (2.3–5.8)	1 (0.3–2.2)	1.3 (0.5–2.7)
	Suspected Emphysema / Asthma	1.7 (0.8–3.2)	1.1 (0.4–2.5)	1.1 (0.4–2.5)
	Non-TB mycobacterial infection	1 (0.3–2.2)	1.7 (0.8–3.2)	1 (0.3–2.2)
	Bronchiectasis	2.1 (1.1–3.7)	2.7 (1.5–4.4)	0.6 (0.1–1.7)
	Pulmonary edema	1 (0.3–2.2)	1 (0.3–2.2)	0.6 (0.1–1.7)
	Broncho vascular inflammation, COPD/ Smoking type	0.4 (0.5–1.4)	1.3 (0.5–2.7)	0.6 (0.1–1.7)
	Non-specific opacification	2.7 (1.5–4.4)	2.9 (1.6–4.7	0.4 (0.5–1.4)
	Suspected Kaposi sarcoma	1.1 (0.4–2.5)	0.4 (0.5–1.4)	0.2 (0.05–1.1)
	Progressive mass fibrosis	0.4 (0.5–1.4)	0	0
	Atelectasis	0.2 (0.05–1.1)	0	0
	Silicosis	0.2 (0.05–1.1)	0	0
	Post radiation fibrosis	0.2 (0.05–1.1)	0	0
Pleura	Pleural effusion/thickening/calcification	11.8 (9.2–14.9)	25.9 (22.2–29.8)	8.8 (6.5–11.5)
	Pleural thickening/calcification: likely benign	7.6 (5.5–10.2)	4.2 (2.6–6.3)	2.7 (1.5–4.4)
	Pleural thickening/calcification: likely malignant	1 (0.3–2.2)	0.2 (0.05–1.1)	0
Mediastinum	Mass, indeterminate, Suspected Lymphoma	1.1 (0.4–2.5)	0.4 (0.5–1.4)	0.6 (0.1–1.7)
	Spinal/para-spinal pathology	1.5 (0.7–3)	0.4 (0.5–1.4)	0.2 (0.05–1.1)
Heart and great vessels	Cardiomegaly	7.2 (5.2–9.8)	2.9 (1.6–4.7)	5 (3.3–7.2)
	Aorta atherosclerosis/elongation	1.1 (0.4–2.5)	6.1 (4.2–8.5)	1 (0.3–2.2)
	Cardiac pathology: Other	0.8 (0.2–1.9)	0.6 (0.1–1.7)	0.4 (0.5–1.4)
	Pericardial effusion	0.4 (0.5–1.4)	0	0
	Pulmonary arterial hypertension (PAH)	0	0.2 (0.05–1.1)	0

*%* Percentage, *CI* Confidence interval, *TB* Tuberculosis, *COPD* Chronic obstructive pulmonary disease

#### Prevalence of non-TB abnormalities by anatomical category

**Fig. 2:**
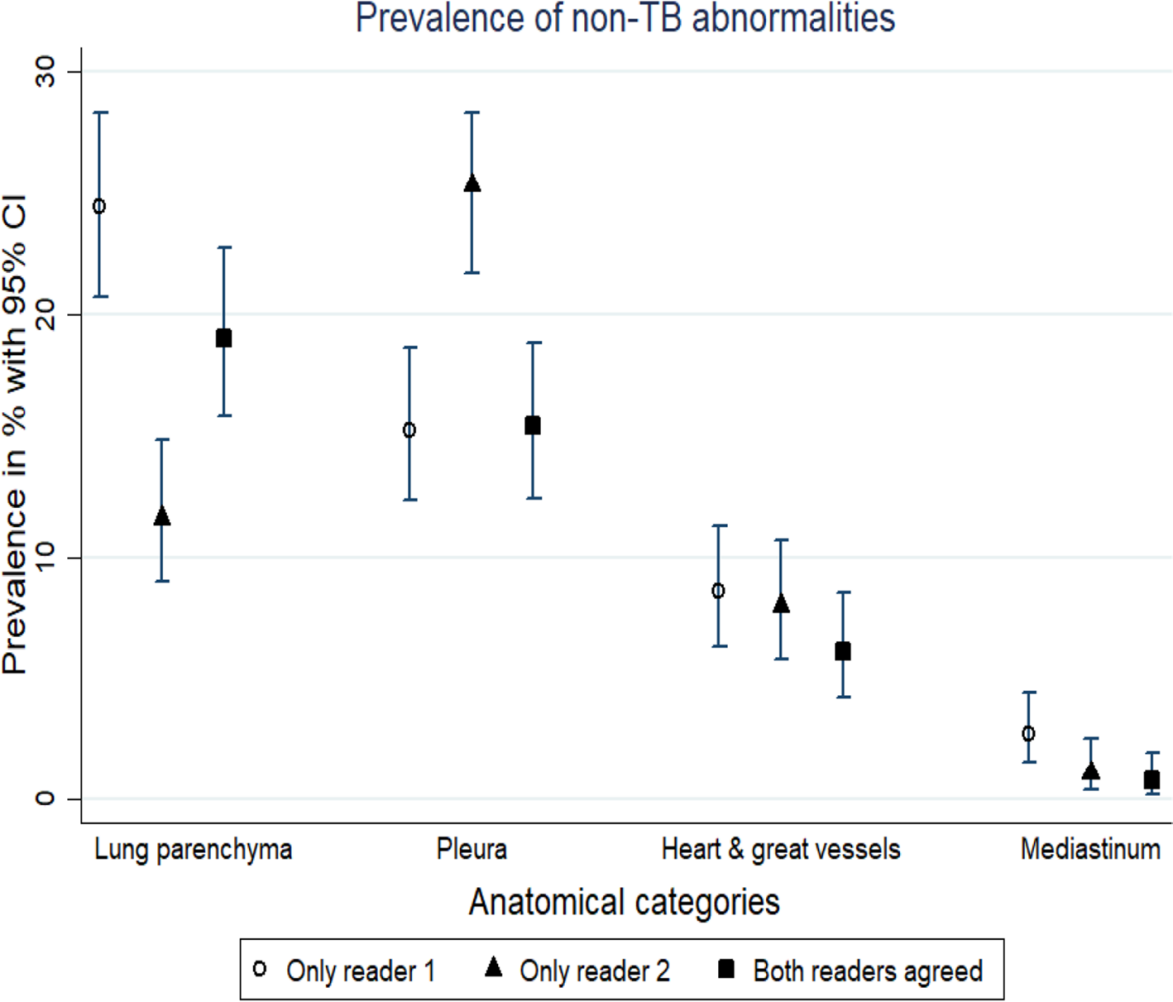
Prevalence of non-TB abnormalities on CXRs with high CAD scores by anatomical categories

Overall, when all non-TB abnormalities were classified into the four anatomical areas based on the location of the abnormality on the digital CXRs, the most common location of non-TB abnormality where both readers agreed was lung parenchyma 19% (95% CI 15.8% - 22.7%). This was followed by pleura 15.4% (95% CI 12.4% - 18.8%), then heart and great vessels at 6.1% (95% CI 4.2%% - 8.5%). Mediastinum had the lowest prevalence of 0.8% (95% CI 0.2% - 1.9%) (Fig. 2).

**Fig. 3:**
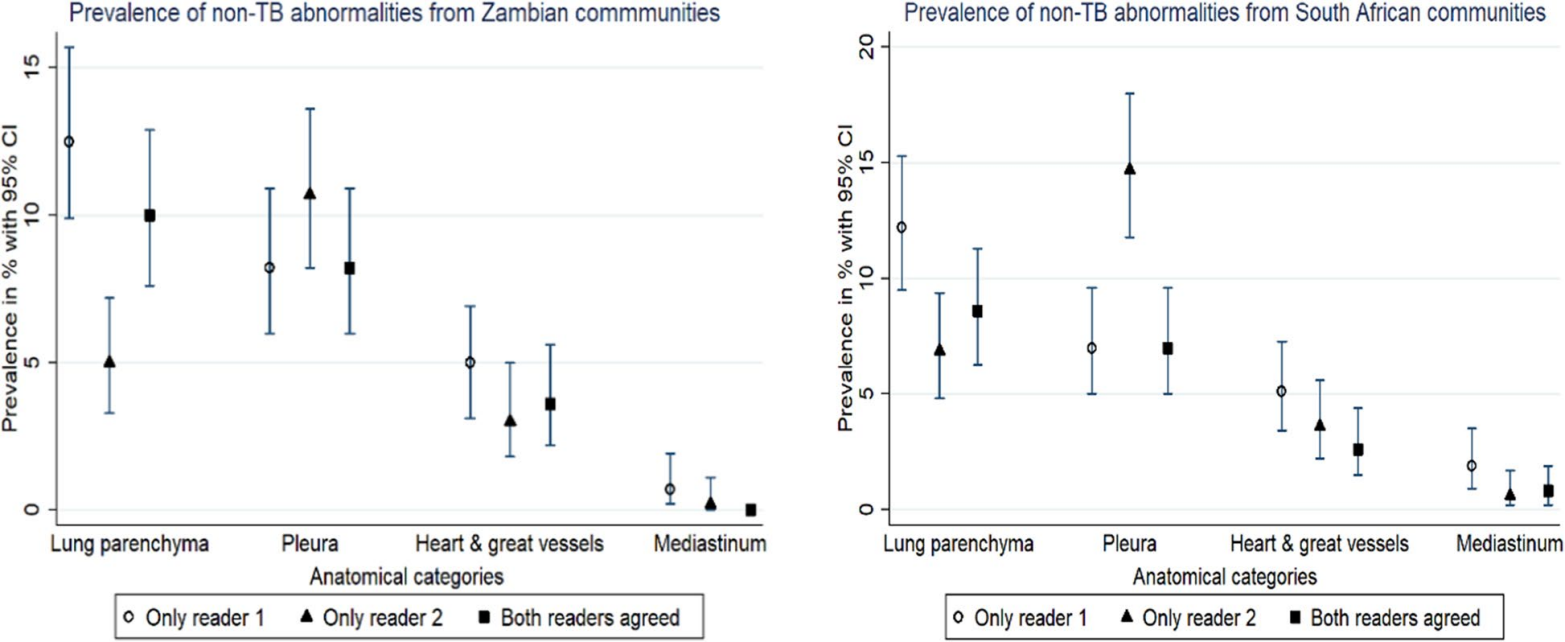
Prevalence of non-TB abnormalities on CXRs with high CAD scores stratified by country

For Zambian communities only, the most common location of non-TB abnormalities where both readers agreed was lung parenchyma 10% (95% CI, 7.6% - 12.9%). This was followed by pleura 8.2% (95% CI, 6% - 10.9%) which was followed by heart & great vessels at 3.6% (95% CI, 2.2% - 5.6%). Mediastinum was the lowest prevalence of 0 (Fig. 3).

Similarly, the most common location of non-TB abnormalities where both readers agreed was lung parenchyma 8.6% (95% CI 6.3% - 11.3%) for SA communities only. This was followed by the pleura 7% (95% CI, 5% - 9.6%) which was followed by heart & great vessels 2.6%, (95% CI, 1.5% - 4.4%). Mediastinum had the lowest prevalence of 0.8% (95% CI, 0.2% - 1.9%) (Fig. 3).

### CAD4TB scores of CXR by anatomical category of non-TB abnormality

**Fig. 4:**
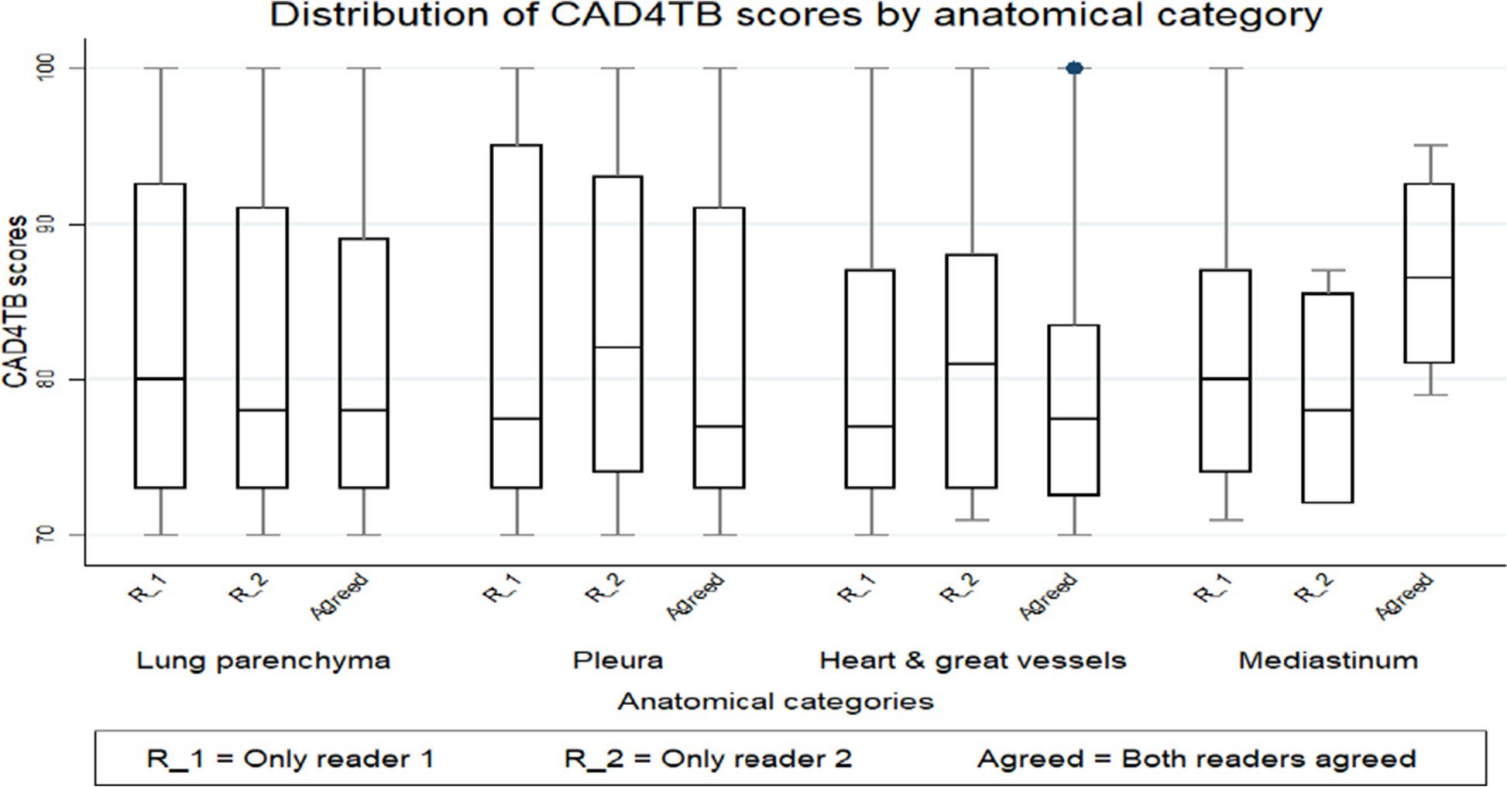
Box plot showing CAD4TB scores for non-TB abnormalities by anatomical category

Where both readers agreed, the lowest median of non-TB abnormalities based on anatomical categories was reported in the Pleura 77 (IQR 73–91). This was followed by ﻿Heart & great vessels 77.5 (IQR 72.5–83.5) then Lung parenchyma 78 (IQR 73–89). The median CAD4TB score for Mediastinum was the highest 86.5 (IQR 81–92.5) (Fig. 4).

## Discussion

This study characterized and estimated the prevalence of non-TB abnormalities on digital CXRs with high CAD4TB scores (≥ 70) from the TREATS TBPS in selected communities in Zambia and South Africa. This is one of the few studies to our knowledge that estimated the prevalence of non-TB abnormalities on digital CXRs with high CAD4TB scores from a TB prevalence survey in Sub-Saharan Africa.

The main findings from this analysis were that a high prevalence and wide range of non-TB abnormalities were identified among individuals with digital CXRs that had high CAD4TB scores with no bacteriologically confirmed TB in the samples submitted, were not on TB treatment and reported no history of TB. These abnormalities included pleural effusions, cardiomegaly, malignant mass nodules, pulmonary edema, pneumonia, interstitial patterns, and many others.

The most common primary non-TB abnormality reported was pleural effusion/ thickening/ calcification at 8.8%. This prevalence was higher than what was reported in a study that was done in Kenya which reported a prevalence of 5.7% for minor pleural effusion/thickening/calcification [15]. Our findings were also higher than what was reported in another study in Malawi which reported a prevalence of 1% for pleural effusions [16]. The current study might have reported a higher prevalence of pleural effusions because Zambia and South Africa are among the top 30 countries with the highest TB burden globally [32] and pleural effusion is a common primary or secondary clinical complication of many disorders including TB, heart failure, bacterial pneumonia, liver cirrhosis, hypoalbuminemia, cancer, emphysema and pulmonary embolism [33, 34]. Early detection and timely referral could improve clinical management of the condition, as high mortality has been associated with pleural effusion especially when it is related to organ failure [35].

In the current study, cardiomegaly was reported at 5% as the second most prevalent non-TB abnormality. This prevalence was much lower than what was found in the Kenya and Malawian studies [15, 16]. The Malawian study reported a prevalence of 20.7% for cardiomegaly while the Kenyan study reported a prevalence of 23.1% for cardiomegaly. A lower prevalence of cardiomegaly reported in the current study could be attributed to the fact that the current study only analyzed data on a sub-group of digital CXRs that had high CAD4TB scores and not all abnormal CXRs irrespective of the CAD scores. Cardiomegaly is indicative of a wide range of underlying cardiovascular conditions such as myocardial infarctions, ischemia, hypertensive diseases, TB pericarditis, effusions, and many more [36]. And generally, a remarkable unanimity in the pattern of heart-related diseases has been documented in African countries [37]. All this coupled with the poor prognosis that is associated with cardiomegaly in adults is suggestive of the importance and necessity of early diagnosis and management of this condition.

The current study also reported a notable prevalence of other non-TB abnormalities that might be of public health relevance like mass/nodules (benign/ malignant) 2.1%. The Kenya study [15] reported a lower prevalence of 0.4% for mass/nodules: malignant and 1.2% for mass/nodules: benign. Over the past two decades, the incidence of cancer has increased dramatically as an emerging publish health problem [38]. Lung cancer is among the most prevalent and leading causes of cancer-related deaths in Southern Africa [39]. Even though the diagnosis of lung malignancy cannot be made entirely on CXRs, detecting suspected malignancy on CXRs with the help of AI might be critical in aiding timely referral to health facilities concerned with cancer management, for early intervention, as the prognosis for cancer worsens with stage [40, 41].

Non-TB abnormalities from the lung parenchyma 19% followed by those on the pleura 15.4% came out as the most prevalent when primary non-TB abnormalities were grouped into 4 major anatomical categories (lung parenchyma, pleura, mediastinum, and heart & great vessels). These findings were different from the Kenya study [15] which reported the heart and great vessels region 26.3% as having the highest group prevalence. These differences reported above could again be attributed to the fact that the current study only analyzed data on a sub-group of digital CXRs with high CAD4TB scores and not all abnormal CXRs.

Generally, the two readers had slight to fair IRA for image reading based on the Cohens’ kappa statistic which ranged from 0.24–0.40 for lung parenchyma, mediastinum, and heart & great vessel abnormalities. Inter-rater agreement of CXRs has been reported to be dependent on the experience of the raters [42, 43]. This is one of the reasons why the study used readers with over 20 years of experience in image reading. However, a long list of items to be read for (the way this study had a long list of non-TB abnormalities) has been reported to reduce IRA [30]. Hence, a fair IRA in the current study was seen as acceptable. On the other side, the readers had higher IRA for images from Zambian communities than South African communities. This could have been caused by the fact that the readers were more used to CXRs in the Zambian context and less used to SA CXRs. Populations are different from the two countries, South Africa has a more dynamic picture of TB, silicosis, and more NCDs (making it more complex to interpret CXRs) [24]. The readers reported on more non-TB abnormalities from SA such as silicosis, progressive mass fibrosis, and post-radiation fibrosis but they did not agree on any of them. Future studies that would look at similar work and aim to improve IRA should consider the possibilities of using a secondary reader to break the tie where primary readers disagree, implementing suitable pilot tests to standardize CXRs abnormality interpretation, and also reducing the number of non-TB abnormalities to be read for based clinical/public health relevance.

Findings that have been reported in this study might be an additional voice on the potential impact of CAD4TB in the diagnostic workflow of non-TB abnormalities. The message to radiologists, clinicians, researchers, and implementors of TBPSs/ACF programs using CAD4TB systems is that, if not TB, efforts can be extended to look at other clinically relevant chest abnormalities in individuals with high CAD4TB scores. This might be necessary for the diagnostic workflow of non-TB abnormalities to better link suspected non-TB-related conditions to appropriate care.

The strengths of this study included blinding the readers from each other’s readings (robustness of the outcome measure). Also, the study used experienced readers with over 2 decades of experience in image reading. The study also used a long list of abnormalities to be read for and still gave the readers an option to add any abnormality that was not on the list. The study also had some limitations. The prevalence of all the non-TB abnormalities was sorely dependent on radiological diagnosis. No other tests were available because initially, this was TB-centered data, aiming to measure TB, and not designed to assess non-TB pathologies. This study could have suffered from reader bias because health facilities do not routinely use digital CXRs to read for all the abnormalities that were reported. Furthermore, some TB cases could have still been missed, leading to the misclassification of CXRs. Lastly, the results for this analysis could only apply to a sub-group of people with high CAD4TB scores but no bacteriologically confirmed TB, not on TB treatment, and no history of TB. Future studies should consider investigating non-TB abnormalities on CXRs with both high and low CAD4TB scores for more comparisons to be made.

## Conclusion

A wide range of non-TB abnormalities (both suspected communicable and NCDs) were identified among individuals that had digital CXRs with high CAD4TB scores but had no bacteriologically confirmed TB in any of the samples submitted, were not on TB treatment, and had no prior history of TB. Computer Aided Detection systems might have the potential to provide information on other non-TB abnormalities that might be of clinical relevance in communities alongside TB. Given the rising burden of communicable and NCDs, it is increasingly becoming necessary for AI systems like CAD/CAD4TB, to have the capability to accurately read for multiple abnormalities such as pleural effusions, cardiomegaly, and masses/nodules (lung cancer) to better link cases to correct care. This might be useful to LMICs where there is no routine screening for non-TB abnormalities and there is often a shortage of qualified radiologists.

### Supplementary Information

**Additional file 1: Table S1.** Baseline characteristics of participants with high CAD4TB scores from TREATS TB prevalence survey.

## Data Availability

The dataset analyzed during the current study is available at ZAMBART, Lusaka Zambia, and from the corresponding author on reasonable request.

## Abbreviations

ZAMBART: Zambia Aids Related Tuberculosis
TB: Tuberculosis
CXR: Chest X-Ray
CAD: Computer-Aided Detection
CAD4TB: Computer-Aided Detection for Tuberculosis
TREATS: Tuberculosis Reduction through Expanded Antiretroviral Treatment and Screening
HIV: Human Immune Virus
AIDS: Acquired Immune Deficiency Syndrome
WHO: World Health Organization
SA: South Africa
ACF: Active Case Finding
AI: Artificial Intelligence
LMIC: Low-to-Middle Income Countries
COPD: Chronic Obstructive Lung Disease
TBPS: Tuberculosis Prevalence Survey
PS: Prevalence Survey
UTT: Universal Testing and Treatment
NTM: Non-Tuberculosis-Mycobacterium
UTH: University Teaching Hospital
IRA: Inter-Rater Agreement
CI: Confidence Interval
MFS: Mobile Field Site
Rx: Treatment
IQR: Inter-Quartile Range
PAH: Pulmonary Arterial Hyperinflation
NCD: Non-Communicable Disease
UNZABREC: The University of Zambia Biomedical Research Ethics Committee
LSHTM: London School of Hygiene & Tropical Medicine
EDCTP: European and Developing Countries Clinical Trials Partnership
