# Supplementary material for: Assessment of non-tuberculosis abnormalities on digital chest x-rays with high CAD4TB scores from a tuberculosis prevalence survey in Zambia and South Africa
Source: BMC Infect Dis. 2023 Aug 8;23:518. doi: 10.1186/s12879-023-08460-0 (PMC10408069; doi:10.1186/s12879-023-08460-0)
Supplement: Supplementary file 1 — Additional file 1: Table S1. Baseline characteristics of participants with high CAD4TB scores from TREATS TB prevalence survey. [file 12879_2023_8460_MOESM1_ESM.docx]

**SUPPLEMENTARY TABLE**

Table S1: Baseline characteristics of participants with high CAD4TB scores from TREATS TB prevalence survey

| **Characteristic** | **Total**  **(N=525)**  **n (%)** | **Country** | |
| --- | --- | --- | --- |
|  |  | **South Africa**  **(N=249)**  **n (%)** | **Zambia**  **(N=276)**  **n (%)** |
| **Sex** |  |  |  |
| Male | 271 (51.6) | 146 (58.6) | 125 (45.3) |
| Female | 254 (48.4) | 103 (41.4) | 151 (54.7) |
| **Age** |  |  |  |
| **(**M/IQR) | 46 (33, 61) | 48 (35,59) | 44 (32, 62.5) |
| **Age group** |  |  |  |
| 15-24 years | 61 (11.6) | 21 (8.4) | 40 (14.5) |
| 25-34 years | 84 (16) | 39 (15.7) | 45 (16.3) |
| 35-44 years | 98 (18.7) | 44 (17.7) | 54 (19.6) |
| 45-54 years | 93 (17.7) | 55 (22.1) | 38 (13.8) |
| 55-64 years | 82 (15.6) | 51 (20.5) | 31 (11.2) |
| 65 years & above | 107 (20.9) | 39 (15.7) | 68 (24.6) |
| **Reported cough** |  |  |  |
| Yes | 84 (16.0) | 23 (9.2) | 61 (22.1) |
| No | 441 (84.0) | 226 (90.8) | 215 (77.9) |
| **Reported weight loss** |  |  |  |
| Yes | 34 (6.5) | 16 (6.4) | 18 (6.5) |
| No | 491 (93.5) | 233 (93.6) | 258 (93.5) |
| **Reported fever** |  |  |  |
| Yes | 26 (5.0) | 14 (5.6) | 12 (4.4) |
| No | 499 (95.0) | 235 (94.4) | 264 (95.7) |
| **Reported night sweats** |  |  |  |
| Yes | 31 (5.9) | 15 (6.0) | 16 (5.8) |
| No | 494 (94.1) | 234 (94.0) | 260 (94.2) |
| **Reported chest pains** |  |  |  |
| Yes | 52 (9.9) | 17 (6.8) | 35 (12.7) |
| No | 473 (90.1) | 232 (93.2) | 241 (87.3) |
| **HIV status** |  |  |  |
| Positive | 83 (15.8) | 30 (12.1) | 53 (19.2) |
| Negative | 285 (54.3) | 137 (55.0) | 148 (53.6) |
| Unknown | 157 (29.9) | 82 (32.9) | 75 (27.2) |
| **History of smoking** |  |  |  |
| Yes | 226 (43) | 159 (63.9) | 67 (24.3) |
| No | 299 (57) | 90 (36.1) | 209 (75.7) |
| **Current alcohol drinkers** |  |  |  |
| Yes | 208 (39.6) | 124 (49.8) | 84 (30.4) |
| No | 317 (60.4) | 125 (50.2) | 192 (69.6) |
| **CAD4TB score** |  |  |  |
| (M/IQR) | 81 (73, 92) | 80 (73,91) | 82 (74, 96) |
| **CAD4TB score category** |  |  |  |
| 70-80 | 259 (49.3) | 128 (51.4) | 131 (47.5) |
| 81-90 | 116 (22.1) | 56 (22.5) | 60 (21.7) |
| 91-100 | 150 (28.5) | 65 (26.1) | 85 (30.8) |

*N= Sample size, n= Frequency, % = Percentage, M/IQR=Median and Interquartile range, TB= Tuberculosis, CAD4TB=Computer aided detection for tuberculosis*
